# Ion Channels of the Sarcolemma and Intracellular Organelles in Duchenne Muscular Dystrophy: A Role in the Dysregulation of Ion Homeostasis and a Possible Target for Therapy

**DOI:** 10.3390/ijms24032229

**Published:** 2023-01-23

**Authors:** Mikhail V. Dubinin, Konstantin N. Belosludtsev

**Affiliations:** 1 Department of Biochemistry, Cell Biology and Microbiology, Mari State University, pl. Lenina 1, 424001 Yoshkar-Ola, Russia; 2 Laboratory of Mitochondrial Transport, Institute of Theoretical and Experimental Biophysics, Russian Academy of Sciences, Institutskaya 3, 142290 Pushchino, Russia

**Keywords:** dystrophin, Duchenne muscular dystrophy, skeletal muscles, cardiomyocytes, ion channels, sarcolemma, sarcoplasmic reticulum, mitochondria, *mdx*

## Abstract

Duchenne muscular dystrophy (DMD) is caused by the absence of the dystrophin protein and a properly functioning dystrophin-associated protein complex (DAPC) in muscle cells. DAPC components act as molecular scaffolds coordinating the assembly of various signaling molecules including ion channels. DMD shows a significant change in the functioning of the ion channels of the sarcolemma and intracellular organelles and, above all, the sarcoplasmic reticulum and mitochondria regulating ion homeostasis, which is necessary for the correct excitation and relaxation of muscles. This review is devoted to the analysis of current data on changes in the structure, functioning, and regulation of the activity of ion channels in striated muscles in DMD and their contribution to the disruption of muscle function and the development of pathology. We note the prospects of therapy based on targeting the channels of the sarcolemma and organelles for the correction and alleviation of pathology, and the problems that arise in the interpretation of data obtained on model dystrophin-deficient objects.

## 1. Introduction

Duchenne muscular dystrophy (DMD) is a recessive X-linked hereditary disease caused by mutations in the DMD gene encoding dystrophin protein [1]. Often these are frame-shifting deletions and insertions that cause premature termination of translation and nonsense-mediated mRNA decay, nonsense mutations, and large deletions in gene regions encoding the N- and C-termini of the dystrophin protein. This is one of the most common forms of muscular dystrophy—DMD is diagnosed in an average of 1 in 3500 boys, extremely rarely clinical manifestations are found in heterozygous girls (<1 per million) [2,3,4]. The pathology demonstrates progressive muscle weakness that manifests itself in early childhood and leads to the inability to walk in the second decade of life. DMD shows damage to the cardiac and smooth muscle, digestive and excretory systems, but skeletal muscle is primarily affected [5]. In addition to progressive muscle weakness, patients experience developmental delay, and breathing and speech problems [6]. More than 90% of male patients aged 18 years with DMD show signs of cardiomyopathy [7,8], most often becoming the main cause of death in the later stages [9,10,11]. There is also a less severe variant of dystrophinopathy—Becker muscular dystrophy (BMD), caused by medium-sized deletions in the middle of the gene that do not affect the reading frame. In this case, many patients retain the ability to move independently even into adulthood [5].

Dystrophin is known to play a key role in linking the muscle cell cytoskeleton (actin microfilaments, intermediate filaments, microtubules, and other related structural proteins) with the sarcolemma, channel proteins, signaling and scaffolding proteins to the extracellular matrix via the dystrophin-associated protein complex (DAPC), maintaining the structural integrity of muscle tissue and its functional activity, and its absence is accompanied by progressive destabilization of the muscle fiber [4,11,12,13]. This is followed by cycles of degeneration/regeneration, inflammation and replacement of muscle tissue with connective and adipose tissue [14]. Along with this, dystrophin and various components of the DAPC are considered as molecular scaffolds to coordinate the assembly of various signaling molecules [15,16]. This includes a wide variety of ion channels and their associated signaling molecules that maintain ion homeostasis and, in particular, stable calcium levels necessary for normal muscle contraction. The important role of dystrophin and DAPC in the regulation of the activity of Na^+^ transporting systems, especially in the heart muscle, is also noted [14,16,17]. Moreover, there is evidence that these proteins are involved in the regulation of potassium ion homeostasis and the functional activity of K^+^ transport channels [16,17]. In the absence of dystrophin and complete DAPC, all these pathways are damaged and contribute to muscle pathology.

Currently, clinical trials are ongoing aimed at creating a gene therapy that allows restoration of the normal expression of dystrophin [18,19], and this can be considered as the only way to truly treat this pathology. However, these approaches often face multiple technical problems, primarily due to vector delivery, and can only be effective in early therapy, before the irreversible replacement of muscle tissue with fibrous deposits. In this regard, much attention is paid to the correction of the above secondary effects of DMD [14]. Perhaps the most common is the use of corticosteroids counteracting inflammation, but they only delay the progression of the disease and show significant side effects [20]. Numerous studies on model animals (mice, rats, golden retrievers, zebrafish, nematodes), which are an approximate pathological model of dystrophinopathies, as well as on biopsy and autopsy tissues from patients suffering from DMD, indicate the possibility of correcting the pathology by normalizing or at least improving ionic homeostasis, leading to an alleviation of the course of the disease.

This review focuses on the role of dystrophin and DAPC in the functioning of ion transport and deposition systems in skeletal and cardiac muscles. We consider the impact of Duchenne dystrophy on the dysfunction of these systems both in the sarcolemma and in intracellular organelles, primarily in the sarcoplasmic reticulum (SR) and mitochondria. Special attention is paid to possible approaches used to improve the functioning of ion homeostasis regulation systems contributing to the correction of pathology.

## 2. Dystrophin and DAPC and Their Role in the Regulation of Sarcolemmal Ion Channels

Dystrophin is a structural cytoskeletal protein encoded by one of the longest genes in the human body called DMD (Xp21.1–Xp22) and spanning over 2.2 Mb in 79 exons and encoding a 14 kb mRNA transcript [21]. There are several variants of dystrophin, each of them is transcribed from different promoters and has different first exon. Full-length Dp427m is predominantly expressed in striated skeletal muscle but also found in smooth muscle and cardiac muscle, Dp427B is found primarily in cortical and cerebellar neurons, and Dp427P is predominantly expressed in Purkinje cerebellar neurons [22]. In addition, the DMD gene may also have internal promoters located at a distance. Each gives rise to truncated dystrophin isoforms such as Dp260 (expressed in the retina), Dp116 (in the peripheral nervous system), Dp140 (in the brain and kidney), Dp71 and the shortest Dp40 (both ubiquitously expressed) [23,24].

**Figure 1:**
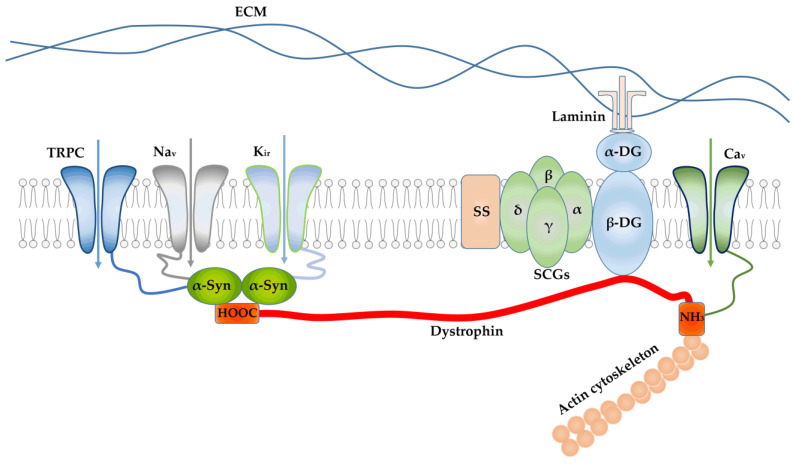
Association of a DAPC with ion channels of the sarcolemma, cytoskeleton, and extracellular matrix. ECM—extracellular matrix, α-DG—α-dystroglycan, β-DG—β-dystroglycan, SCGs—sarcoglycans, α-Syn—α-syntrophin, SS—sarcospan, TRPC—transient receptor potential channel, Ca_V_—voltage-gated calcium channel, Kir—inward-rectifier potassium channel.

In muscles, dystrophin is localized on the cytoplasmic surface of the sarcolemma (Figure 1). A full-sized dystrophin molecule consists of four domains [25]. The N-terminal domain contacts actin (mainly with filamentous γ-actin). Dystrophin is an anchor protein for actin and is therefore involved in the control of deformation of the cell membrane surface. It is followed by a central domain containing 24 spectrin-like repeats and 4 hinge regions, has a strongly elongated shape, and, since each spectrin-like repeat consists of three α-helices, is sufficiently flexible and elastic. Following the central domain, there is a cysteine-rich domain, which connects the muscle fiber cytoskeleton with the extracellular matrix, and also contacts with the calcium channels of the sarcolemma. Finally, the last C-terminal domain binds dystrobrevins and syntrophins. Dystrophin is part of the costameric DAPC responsible for the connection of actomyosin complexes (sarcomeres), the plasma membrane of the muscle fiber (sarcolemma), and extracellular matrix proteins, thus performing an anchor function. In addition to dystrophin, DAPC includes α and β dystroglycans forming the dystroglycan subcomplex, and sarcoglycans (α, β and γ) forming the sarcoglycan subcomplex, as well as sarcospan, syntrophins (α, β1 and β2), dystrobrevins (α1, α2, α3), and neuronal nitric oxide synthase (nNOS) and other components. In fact, dystrophin, interacting with other components of DAPC (Figure 1), serves as a molecular shock absorber that protects the sarcolemma and muscle fiber from damage during muscle contraction and relaxation.

Along with this, dystrophin in combination with other DAPC elements acts as molecular scaffold to coordinate the assembly of various signaling molecules including ion channels and regulate mechanosensitive ion channels, and is central to mechanotransduction at the costamere [15,25,26]. In particular, through α-syntrophin, DAPC interacts with ion channels of the sarcolemma, namely, calcium channels TRPC1 and TRPC4, sodium channels Na_v_1.4 and Na_v_1.5, and potassium channels Kir2 and Kir4.1 [15]. The functioning of these channels is significantly impaired in the absence of dystrophin (in the case of DMD or BMD) and other pathologies associated with the loss of DAPC components [16,27].

### 2.1. Dysregulation of Sarcolemmal Calcium Channels in DMD

Abnormal calcium homeostasis is perhaps one of the most important consequences of loss of DAPC integrity. Transcriptomic analysis of DMD muscle samples reveals changes or activation of genes involved in calcium homeostasis [28]. This is expressed in the excessive entry of calcium ions from the extracellular matrix into muscle cells and is detected already at early stages of the disease. Indeed, dysregulation of calcium homeostasis was detected in muscle fibers of DMD boy fetuses and measured in not fully differentiated human DMD myotubes [29,30].

**Figure 2:**
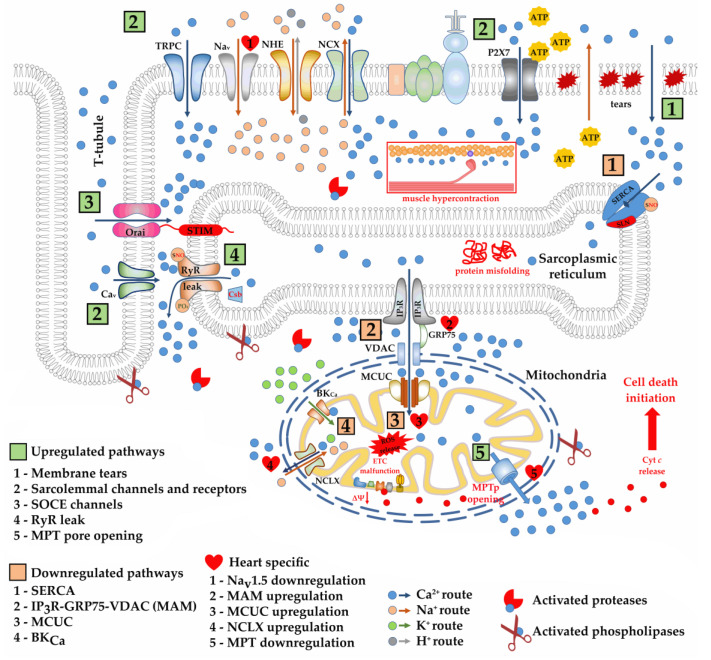
Schematic representation of the features of the functioning of the ion transport pathways of the sarcolemma, sarcoplasmic reticulum, and mitochondria in dystrophin-deficient striated muscle and the consequences of these changes. The picture shows upregulated and downregulated pathways. Specific changes identified in the heart in the early stages of DMD are also highlighted.

Excessive intake of calcium into muscle cells is considered to be due to the appearance of microtears [31,32,33] in the sarcolemma (Figure 2) during mechanical stress, which is accompanied by an increase in the permeability of the sarcolemma for ions. At the same time, it was shown that mechanical ruptures of the sarcolemma of muscle fibers in dystrophin-deficient *mdx* mice are able to recover quickly [34], so the contribution of this pathway to the dysregulation of ion homeostasis is rather difficult to assess. However, it should be noted that the use of membrane sealants such as Poloxamer 188 and its derivatives has been shown to improve cardiac and skeletal muscle function in dystrophin-deficient mice and dogs in a number of studies [35,36]. However, other studies show no positive effect of this approach [37,38].

On the other hand, it is believed that excessive calcium entry into the muscle fiber is associated with dysfunction of the calcium-transporting channels of the sarcolemma, represented by transient receptor potential channels (TRPC), voltage-gated calcium (Ca_V_) channels, P2X7 purinoceptors, and store-operated Ca^2+^ entry (SOCE) channels (Figure 2). Sarcolemmal TRPCs are activated by Ca^2+^ store depletion and/or membrane stretch and are considered stretch-activated channels. TRP and in particular C1, C3, and C6 are overexpressed in the skeletal, cardiac, and smooth muscles of dystrophin-deficient *mdx* mice [39,40,41]. Until recently, the most studied was the TRPC1 calcium transport pathway formed with tyrosine–protein kinase Src and caveolin-3 [40]. It interacts with DAPC, namely dystrophin and α1-syntrophin serving as scaffolds for signaling molecules [42]. The loss of dystrophin and DAPC integrity in DMD makes these channels more sensitive to mechanical activation [43]. In the case of TRPC6 it is accompanied by post-translational modifications and, in particular, phosphorylation by Ca^2+^ calmodulin-dependent kinase (CaMKII) [44] or extracellular response kinase (ERK1/2) [45] leading to the activation of TRPC6 and promoting an increase in the absorption of extracellular calcium. TRPC6 levels are known to be elevated in cardiac and skeletal muscle in mice [41,46,47], and humans in DMD [48,49]. It has recently been shown that genetic or pharmacological inactivation of TRPC6 with the small molecule BI 749327 results in improving skeletal and cardiac morphology and dysfunction, and reducing skeletal and bone deformities in mice lacking both dystrophin and utrophin (*mdx/utrn^–/–^* double knockout (dko) mice) representing a severe DMD model [50]. This confirms the involvement of TRPC6 in the development of striated muscle pathology. In addition, it was also recently found that the skeletal muscles of DMD rats show an increase in TRPC3 levels already at 1.5 months of age, while TRPC1 levels significantly increase only at the 7th month of animal development, but their localization did not change [51]. This led to the conclusion that the calcium overload of muscle fibers is initially due to overexpression and an increase in TRPC3 activity, presumably by unglycosylation, which is known to enhance the activity of that channel [52]. This is also confirmed in experiments with a specific inhibitor of TRPC3 Pyr10 reducing the permeability of the sarcolemma for calcium and mitigating the development of the dystrophic process [51]. TRPC3 is also involved in the development of muscular dystrophies. Overexpression of this channel induced muscle dysfunction in healthy wild-type mice, independent of dystrophin-related membrane tears [53].

It can also be suggested that TRPC4 is involved in the disruption of calcium transport through the sarcolemma in DMD, although there is no direct evidence for this. TRPC4 is associated with DAPC, namely α-syntrophin and its knockdown has been shown to lead to an abnormal increase in calcium uptake via TRPC1 and TRPC4 [42]. In addition, Vandebrouck et al. [54] showed that antisense downregulation of TRPC1 and TRPC4 reduced the SOCE in *mdx* fibers.

A number of publications also note the role of another channel belonging to the TRPC family, known as transient receptor potential vanilloid type 2 (TRPV2), in increasing the level of intracellular calcium in Duchenne dystrophy. Normally, TRPV2 is localized in the intracellular membrane compartments. However, in the case of DMD, TRPV2 translocates to the sarcolemma, as shown in skeletal muscles from human patients with muscular dystrophy, δ-sarcoglycan-deficient BIO14.6 hamsters, and *mdx* mice [55,56]. Moreover, an increase in TRPV2 was also noted in the BIO14.6 hamster cardiomyocyte sarcolemma, but was not observed in young (5–8 weeks) *mdx* mice [55]. In this case, overexpression of a dominant negative variant of TRPV2 mitigated the development of pathology in the studied animal models [56]. At the same time, cardiomyocytes of old *mdx* mice (10–12 months old) displayed a significant TRPV2 localization at the sarcolemma, which promotes a massive influx of calcium into the cell, especially under enhanced stretching conditions [57].

Another mechanism for calcium entry into muscle cells from the extracellular space is due to voltage-gated calcium (Ca_V_) channels activated in response to membrane depolarization (Figure 2). Cardiomyocytes of 4–6 month old *mdx* mice showed an increase in the activity of L-type Ca_V_ and Ca^2+^ influx during the action potential, although the level of protein and mRNA of this channel did not change [58]. This, according to the authors, contributes to abnormalities in the electrophysiology of the heart muscle and arrhythmia. Inhibition of L-type Ca_V_ by diltiazem and verapamil has been shown to reduce the rate of necrosis in *mdx* mice [59]. A similar result was obtained in the case of nifedipine treatment [60]. However, it is important to note that no positive effect of L-type Ca_V_ inhibitors has been observed in DMD patients.

Overactivation and overexpression of P2X7 sarcolemmal purinoceptors in response to high concentrations of extracellular ATP observed in dystrophin-deficient muscles of *mdx* mice can also be noted as additional mechanisms for excess calcium entry into muscle cells (Figure 2) [61,62]. In this case, a reduction in the level of extracellular ATP, as well as genetic and pharmacological blockade of P2X7, improved the parameters of calcium entry in dystrophic muscles [63,64,65].

The SOCE mechanism of extracellular Ca^2+^ entry into muscles deserves special mention. SOCE channels of the sarcolemma responsible for this mechanism are activated in response to the depletion of intracellular Ca^2+^ depots and, first of all, the SR, and is aimed at replenishing intracellular calcium stores in depot organelles [66]. The involvement of mitochondria, which are also intracellular calcium depots, in the regulation of SOCE is also discussed [67]. Abnormal calcium overload in DMD is also due to disruption of SR and mitochondrial ion channels promoting the release of calcium from these depots and, in turn, leads to chronic activation of SOCE channels [68]. The SR membrane contains Ca^2+^ sensor STIM1 (stromal interaction molecule), self-oligomerizing in response to calcium depletion and activating SOCE by binding to Ca^2+^ channels in the sarcolemma (Figure 2) [69,70,71]. Orai1, which is highly selective for calcium, is usually considered as this channel. STIM1-Orai1 expression and activity are known to be elevated in DMD and contribute to further muscle calcium overload [72,73]. This has been shown both in *mdx* mice and biopsy specimens from DMD patients [74,75]. Moreover, STIM1 overexpression is known to promote muscle dystrophy in healthy mice and this effect is blocked by crossing in a transgene expressing a dominant-negative Orai1 (dnOrai1) mutant [74]. Pharmacological blocking of SOCE via STIM1-Orai1 in myotubes from DMD-patient-derived induced pluripotent stem cells also prevented Ca^2+^ overload and restored contractility [75]. In addition, breeding transgenic mice expressing the dominant-negative mutant Orai1 (dnOrai1) with *mdx* mice resulted in a significant improvement in mouse skeletal muscle histopathology, further supporting the involvement of STIM1-Orai1 in the progression of DMD [74]. A recent study also showed that crossing *mdx* mice with muscle-specific Orai1 knockout *mdx* mice (*mdx*-Orai1 KO mice) also resulted in normalizing Ca^2+^ homeostasis and promoting sarcolemmal integrity/stability [76].

TRPC channels of the sarcolemma can also be considered as SOCE channels. It is shown that in this case SOCE can be implemented through interaction with STIM1 [77,78]. The activity of TRPC1 is known to be higher in dystrophic myotubes from *mdx* mice and DMD patients, which may also contribute to a more active SOCE [54,79,80,81].

### 2.2. Dysregulation of Sarcolemmal Sodium Channels in DMD

The development of DMD is closely related to intracellular sodium overload. DAPC, via α-syntrophin, is also involved in the scaffolding of voltage-gated sodium (Na_V_) channels of the sarcolemma (Na_V_1.4 in skeletal muscle and Na_V_1.5 in the heart) [82] and loss of complex integrity also leads to dysfunction of these channels (Figure 2). Skeletal muscles of *mdx* mice show an increase in the conductive properties of Na_V_1.4 channels contributing to increased inward Na^+^ current [83]. Interestingly, both gene expression and Na_V_1.4 protein level were reduced in the muscles of *mdx* mice. Inhibition of its activity with tetrodotoxin led to the normalization of the influx of Na^+^ in *mdx* muscle to the wild-type level and the restoration of the state of the muscle fiber [83]. On the contrary, cardiomyocytes of *mdx* mice demonstrate a decrease in sodium current associated with a twofold reduction in the level of the Na_V_1.5 protein playing a key role in the excitability and conduction of the heart [84]. This, according to the authors, contributes to cardiac electrophysiological abnormalities and the development of cardiomyopathy.

Skeletal muscles of dystrophin-deficient BIO14.6 hamsters and *mdx* mice also show an upregulation of Na^+^–H^+^ exchanger (NHE-1) activity contributing to sodium overload (Figure 2) [85,86]. In this case, NHE inhibitors, cariporide, and 5-(N-ethyl-N-isopropyl)-amiloride (EIPA) show protective effects against muscle degeneration in both model animals [85]. Selective inhibition of NHE by rimeporide has also been shown to have beneficial effects on the state of skeletal and cardiac muscles in dystrophin-deficient hamsters, mice, and golden retrievers [87,88].

It is important to note that an increase in Na^+^ levels also secondarily contributes to an increase in calcium levels in muscle fibers due to overactivation of the Na^+^/Ca^2+^ exchanger (NCX) (Figure 2). In human myotubes from DMD patients, NCX works in a reverse mode, pumping out excess sodium, but this is accompanied by an additional influx of calcium ions into the myoplasm [89].

### 2.3. Dysregulation of Sarcolemmal Potassium Channels in DMD

One could assume that the features of potassium transport in DMD are the least studied. This is especially true for skeletal muscles and the heart, whose ion channels are the subject of this review (significant changes in the functioning of the brain and retina Kir channels are also described in [16]). In a number of studies, including the earliest ones, no changes were found in outward K^+^ currents in cardiomyocytes of *mdx* mice [58,90], whose potassium channels are the main determinants of the resting membrane potential and action potential repolarization. However, cardiomyocytes of dystrophic dogs show a reduction in transient outward K^+^ currents [91], which, according to the authors, also contributes to the imbalance of inward and outward currents in the dystrophic myocardium and the development of cardiopathology. In the case of inward rectifier K^+^ currents, mainly mediated by Kir2.1 channels, a reduction was noted in *mdx* mouse cardiomyocytes, which also correlates with a decrease of sodium currents through Na_V_1.5 channels [92], also associated with α-syntrophin of the DAPC. However, if Na_V_1.5 channels showed a decrease in the protein level in cardiomyocytes of *mdx* mice [84], then the level of Kir2.1 and its localization did not change [92] suggesting the participation of other inwardly rectifying potassium channels in the decrease in potassium fluxes in dystrophic cardiomyocytes.

We can also note the data on the involvement of cardiomyocyte K_ATP_ channels in the development of DMD. K_ATP_ channels are metabolic sensors that regulate cell activity according to metabolic status [93]. K_ATP_ subunit Kir6.2 is shown to be associated with dystrophin, as well as with creatine kinase muscle isoform (CK_m_) acting as a regulator of K_ATP_ activity [94]. Therefore, the absence of dystrophin and loss of functional association with CK_m_ in *mdx* mice results in a disability of K_ATP_ in sensing the intracellular ATP concentration and is accompanied by a significant decrease in K_ATP_ currents in cardiomyocytes [94]. The decrease in K_ATP_ channel activity is known to be closely associated with the development of various forms of cardiomyopathy [95,96] and, apparently, also contributes to the development of cardiac pathology characteristic of the late stages of DMD. At the same time, in the case of skeletal muscles, it is noted that distribution, the conductance properties, and ATP sensitivity of K_ATP_ channels do not differ in wild-type and in *mdx* animals [97].

In addition, another potassium channel, the high conductance Ca^2+^-activated K^+^ channel (BK_Ca_ channel), present in the sarcolemma, as well as in the nuclear and mitochondrial membranes, deserves attention. The activity of this channel in skeletal muscles was studied to assess the subsarcolemmal calcium concentration in *mdx* mice. It was shown that the unitary conductance, the calcium- and the voltage-sensitivity did not differ in the muscles of young *mdx* and wild-type mice [98].

Perhaps the most detailed study of the role of the BK_Ca_ channel in the development of DMD has been studied using the nematode *Caenorhabditis elegans* expressing orthologues of the mammalian BK_Ca_ channels known as Slo-1 and Slo-2 channels [99,100]. Dystrophin has been shown to be required for proper localization of Slo-1 in *C. elegans* [101] and, apparently, for its normal functioning. These channels have been found to be located near areas rich in calcium and, in particular, near L-type calcium channels, which is necessary for their timely stimulation, leading to potassium outflow and weakening of muscle hyperexcitation and hypercontraction in response to a significant increase in calcium content. Mislocalization of Slo-1 leads to disruption of these relationships [101].

## 3. Consequences of Impaired Sarcolemmal Ion Permeability in DMD

The sarcolemma of dystrophin-deficient muscles and its channels lose their ability to adequately regulate the main ion currents (Figure 2), which is necessary for correct muscle contraction and relaxation. Primarily, this is based on the excess intake of calcium ions directly controlling the excitation–contraction coupling [102,103]. This is caused both by membrane ruptures and overactivation of TRPC and P2X7 purinoceptors pumping calcium directly into the muscle cell. Moreover, this seems to be facilitated by the overactivation of sodium entry through sodium channels, which, due to the activation of sodium–calcium exchange via NCX, leads to an additional influx of calcium from the extracellular space. Finally, excess sodium causes depolarization and chronic activation of the L-type calcium channels, leading to the release of calcium from the largest intracellular depot, the sarcoplasmic reticulum, via the ryanodine receptor (RyR), and also from the mitochondria via Na^+^-Ca^2+^-Li^+^ exchanger (NCLX). The depletion of intracellular organelles also leads to the activation of SOCE channels, which attempt to replenish the level of calcium in the SR, but additionally contributes to calcium overload of muscle cells. Massive pumping and failure to buffer excess calcium prevent muscle fibers from being able to relax. Indeed, hypercontraction and deficit in muscle relaxation are specific DMD features across species [104]. One could assume that a decrease in potassium currents, which are major determinants of the resting membrane potential and action potential repolarization [17], also contributes to the disturbance of excitation and relaxation. This is especially important for the heart, whose potassium channels maintain the rhythm of contractions by repolarizing the cardiomyocytes so that the electrical and contractile mechanisms remain synchronized [105,106]. In the case of DMD, this appears to contribute to the development of arrhythmia and cardiomyopathy.

Along with the violation of the excitation–contraction coupling, the excess of ions in the myoplasm has other consequences. We can note the violation of muscle cell differentiation [107], depending on calcium homeostasis. In addition, DMD shows overactivation of Ca^2+^-dependent proteases known as calpains causing massive proteolytic damage to cellular proteins [108,109], as well as Ca^2+^-dependent phospholipases disrupting cell and organelle membrane structure and packaging (Figure 2) [110]. These processes are followed by the development of inflammation and the replacement of muscle tissue with connective and adipose tissue (fibrosis) [4]. It is also known about ectopic calcification in the skeletal muscles and diaphragm of *mdx* mice, accompanied by the formation of calcific deposits and contributing to the loss of functional tissue [111,112]. In addition, cytoplasmic sodium overload also causes severe osmotic oedema in DMD patients [113].

Finally, excessive entry of ions into the cell causes dysfunction or, in some cases, an adaptive response of intracellular organelles—the main internal regulators of ionic homeostasis, and in this case, first of all, we are talking about the sarcoplasmic reticulum and mitochondria. On the one hand, this manifests itself in further dysregulation of ionic homeostasis. On the other hand, this also leads to metabolic dysfunction and induction of cell death pathways, since mitochondria are the main producers of ATP, which is necessary for muscle contraction, and also contain factors whose release leads to the initiation of various cell death pathways [114,115].

## 4. The Role of Ion Channels of Intracellular Organelles in the Development of DMD

DMD is well known to show a significant change in the functioning of intracellular organelles. Indeed, dystrophin, through its multiple protein connections, plays an important role in the regulation of signaling and delivery of various molecules, including those to intracellular organelles that regulate intracellular ion homeostasis and, in particular, to the sarcoplasmic reticulum and mitochondria. On the one hand, the loss of dystrophin leads to disruption of the correct functioning of the cell cytoskeleton ensuring the functional relationship of organelles and sarcomeres. On the other hand, disruption of the sarcolemmal channels causes a significant change in the flow of various ions into the muscle cell from the extracellular matrix, which can both induce organelle dysfunction leading to cell death and, as a response, cause reprogramming of the ion and metabolite transport systems in them and, in the case of mitochondria, this leads to changes in the bioenergetics of the muscle cell. The well-known effect of reactive oxygen species (ROS) and reactive nitrogen species (RNS) hyperproduction observed in DMD on the state of transport systems of intracellular organelles should also be noted [26].

### 4.1. SR Channel Abnormalities in DMD

The sarcoplasmic reticulum acts as the main depot of calcium ions in the muscles, maintaining the correct dynamics of this ion, which is necessary for the correct regulation of muscle contraction–relaxation cycles [116]. According to various estimates, depending on the specific striated muscle, about 70–90% of the calcium necessary for contraction is released from the SR cisterns [117,118]. In DMD, the transport systems responsible for the uptake and buffering of calcium, as well as the timely release of the ion into the myoplasm, are significantly altered contributing to the uncoupling of muscle contraction and relaxation.

Calcium uptake into the SR lumen is mediated by sarco/endoplasmic reticulum Ca^2+^-ATPase (SERCA) and corresponds to the muscle relaxation phase. The effectiveness of SERCA functioning has been shown to be significantly reduced in dystrophic skeletal muscles and cardiomyocytes contributing to a chronic increase in the level of the ion in the myoplasm (Figure 2). This often does not correlate with the level of different SERCA isoforms in various muscle types. In particular, SERCA1a expression is downregulated in the EDL muscle *mdx* mice but upregulated in the spared intrinsic laryngeal and toe muscles [119,120]. SERCA2a is elevated in the fast-twitch muscles of *mdx* and *mdx:utr^−/−^* mice, as well as in the extensor carpi ulnaris muscles of the canine DMD model [119,121]. At the same time, in the latter case, the level of SERCA1a does not change [109]. Interestingly, in the case of the *mdx:utr^−/−^* mouse and DBA/2J *mdx*, SERCA activity is much more suppressed than in the case of the classical C57 *mdx* mouse [109,121,122], and this, apparently, contributes to a more pronounced calcium overload and, as a result, development of dystrophy. At the same time, gastrocnemius DBA/2J *mdx* mice show an increase in the expression of SERCA1 and SERCA2, which, as in other cases of increased activity of SERCA isoforms, may indicate an adaptive response aimed at improving SR Ca^2+^ handling, but this, apparently, is not enough [122]. Along with this, transgenic overexpression of SERCA1 in skeletal muscles of *mdx* and *mdx:utr^−/−^* mice improved the pathology [123,124]. It is also important to note that there were no differences in SERCA activity and SERCA2 levels in the left ventricles of young DBA/2J *mdx* and C57 *mdx* mice and their respective wild types [122], which is also consistent with the known data on the later development of cardiac impairments both in these mice and patients suffering from DMD. In addition, adeno-associated virus (AAV)-mediated overexpression of SERCA2 in aged *mdx* mice improved cardiac electrophysiology [125] and, in the case of young animals, significantly slowed down the development of cardiomyopathy [126].

One could assume that SERCA undergoes post-translational modifications, which is typical for many systems in DMD and is primarily due to the overproduction of ROS and RNS. SERCA is known to contain sites susceptible to nitrosylation and nitration induced by RNS, ultimately impairing their ability to transport Ca^2+^ [127,128,129,130]. Indeed, skeletal muscles and left ventricle of D2 *mdx* mice show an increase in total nitrocysteine and nitrotyrosine levels, reflecting signs of early onset oxidative/nitrosative stress [122] and may impair SERCA’s ability to adequately uptake calcium ions.

Another factor that has a significant, possibly decisive, effect on the ability of SERCA to uptake calcium is a change in the expression of small peptides that finely modulate its activity. These factors include the following major inhibitors: phospholamban (PLN) reducing mainly SERCA2a affinity for Ca^2+^ in the ventricles [131,132]; myoregulin (MLN) performing the same function in skeletal muscles [133]; sarcolipin (SLN) acting both on SERCA 1 and 2 isoforms of skeletal muscles and atria, and uncoupling and ATP hydrolysis from Ca^2+^ transport, and is also thought to be functionally related to body metabolism and heat production [131,132,134,135]. The dwarf open reading frame (DWORF) is considered as a universal activator of various SERCA isoforms [136]. The level of PLN in the ventricles DBA/2J *mdx* and C57 *mdx* is reported to be unchanged compared to wild-type [122]; however, elimination of PLN exacerbates *mdx* cardiomyopathy [137]. In turn, SLN was found to show a significant increase in expression in skeletal muscles of various mouse models (higher in dko mice), as well as in cardiac muscle, contributing to the suppression of SERCA [109,121]. The same data were obtained on dystrophic dogs [107] and DMD patients [109]. This is also thought to contribute to the disruption of myogenic differentiation in DMD [107]. In this case, knocking out or suppressing SLN improves skeletal and cardiac muscle health/function in *mdx* models and increases the lifespan of the animals [109,138,139,140]. However, it is also noted that the genetic deletion of SLN, on the contrary, aggravates the course of the disease, limiting the activation of calcineurin [141], which counteracts dystrophic pathology [142,143]. It can also be noted that overexpression of the SERCA DWORF activator prevents heart failure in a mouse model of dilated cardiomyopathy [144]. This strategy needs further testing in dystrophin-deficient animal models that also demonstrate the development of cardiomyopathy.

Calcium release from the SR is mediated by the ryanodine receptor (RyR) during a muscle contraction session. This process occurs through direct interaction of various RyR isoforms with L-type Ca^2+^ channels (Ca_V_1.1) on the exterior membrane that are located on the transverse tubules (T-tubules) [145]. RyR has been shown to become leaky in skeletal muscles and heart in DMD (Figure 2) [146,147,148,149]. In the skeletal muscles of *mdx* mice, this is due to post-translational modifications of RyR1 and, in particular, S-nitrosylation by inducible NOS (iNOS) [146] or, according to other authors, by neuronal NOS (nNOS) [147]. A similar process with the participation of RyR1 is also observed in the heart muscle and causes cardiac arrhythmias in *mdx* mice [148]. Along with this, there is a depletion of the critical RyR regulator calstabin maintaining the closing state of RyR both in skeletal muscles [146] and in the heart [148] of *mdx* mice, which also contributes to RyR calcium leakage. Moreover, *mdx* mouse hearts show RyR2 phosphorylation and oxidation also contributing to RyR malfunction [148,149] and, in this case, genetic inhibition of RyR2 phosphorylation prevents its oxidation [150]. Treatment with Rycal improving binding of calstabin to RyR, also prevented SR Ca^2+^ leak and improved skeletal muscle calcium homeostasis in *mdx* mice [151], as well as induced pluripotent stem-cell-derived diseased cardiomyocytes [152]. A recent study by Cleverdon and colleagues shows that skeletal muscle of young DBA/2J *mdx* and C57 *mdx* show a decrease in RyR1 expression compared to wild-type animals, and there is also a decrease in calstabin levels in the diaphragm, contributing to impairments in SR Ca^2+^ uptake. At the same time, the authors did not reveal any changes in RyR2 expression and the level of regulatory factors in the heart of dystrophic animals [122]. Another mechanism for calcium release from the SR is associated with the activity of the inositol 1,4,5-trisphosphate receptor (IP_3_R). IP_3_R also shows changes with the development of DMD and we will look at this in more detail in Section 4.3 concerning mitochondria-associated membranes (MAM contacts) enriched with IP_3_R. A number of reports (summarized in [153]) also note changes (or lack thereof) in the various SR luminal resident proteins buffering Ca^2+^ concentrations and regulating SR Ca^2+^ uptake and release, and also contributing to the development of calcium dysregulation both in the SR and in the muscle cell as a whole.

Importantly, nothing is known about the effect of DMD on the function of other ion channels localized in the SR membrane. First of all, we are talking about the channels that maintain the electrical charge balance across the SR membrane, which is necessary for the correct transport of calcium [154,155]. These include potassium permeable trimeric intracellular cation channels (TRIC channels), ATP-sensitive and Ca^2+^-activated potassium channels (K_ATP_ and BK_Ca_ channels, respectively), and potassium–hydrogen exchanger (KHE). Moreover, the RyR mentioned above is also considered to transport K^+^, having its own countercurrent [156]. These channels, in some cases, are also capable of transporting other ions (Na^+^, Mg^2+^, or H^+^). It is assumed that along with charge-compensating these channels also control SR lumen volume, thereby modifying SR functional properties and, above all, Ca^2+^ handling capacity [155]. In particular, TRIC-KO mice show impaired SR Ca^2+^ transport and embryonic heart failure [157]. Given the versatility of the molecular mechanisms of DMD development, it is possible that the functioning of these channels can also be compromised in this pathology and requires investigation.

### 4.2. Mitochondrial Ion Channels in DMD

Mitochondria being the powerhouse of cells generate the majority of ATP, including the necessary for the functioning of the contractile apparatus of striated muscles. Along with this, mitochondria are direct participants in the cellular homeostasis of calcium, potassium, and other ions. In this regard, these organelles are involved in the regulation of various signaling pathways that initiate or prevent cell death.

Mitochondria are surrounded by two membranes. The outer membrane is freely permeable to ions and most compounds due to the presence of voltage-dependent anion channels (VDAC) that interact with the cell cytoskeleton through microtubules [158]. In the skeletal muscles of D2.*mdx* mice, demonstrating a violation of this interaction due to the absence of dystrophin, there was a violation of membrane permeability to ADP/ATP turnover [159]. It is also important to note that the loss of cytoskeletal architecture in the mdx ventricular myocytes is accompanied by a disruption in the functional relationship between L-type Ca^2+^ channels and VDAC of mitochondria, contributing to a decrease in membrane potential and suppression of the metabolic activity of organelles. In this case, VDAC blocking restored the mitochondrial membrane potential [160].

The inner membrane of mitochondria contains many specific ion channels that provide selective permeability for ions and metabolites. The emphasis of most modern studies is based on the role of calcium transport dysregulation in mitochondria, which is generally not surprising and is due to the role of this ion in the pathogenesis of DMD, as well as the sufficient advancement of techniques for measuring the fluxes of this ion in organelles, cells, and tissues. In addition, the last decade has been marked by important discoveries related to the identification of the molecular structures of many mitochondrial Ca^2+^ transporting systems. In particular, a multisubunit complex of mitochondrial calcium uniporter (MCU) [161,162], Na^+^/Ca^2+^ exchanger (NCLX) [163], and Ca^2+^/H^+^ exchanger [164] was discovered. Finally, we can note the background to understanding the structure and regulation of an important phenomenon known as the mitochondrial permeability transition pore (MPTp) [165,166].

The main pathway for Ca^2+^ uptake through the inner mitochondrial membrane is the Ca^2+^ uniporter complex (MCUC), consisting of transmembrane channel subunit MCU and its dominant negative paralogue MCUb, as well as MICU1 and MICU2 giving it Ca^2+^ sensitivity, and regulatory subunits EMRE, MCUR1, etc. [165]. We have recently shown that the intensity of Ca^2+^ uniport is significantly reduced (Figure 2) in the skeletal muscle mitochondria of *mdx* mice, and this is already characteristic of young 4 week old animals and persists at least up to 7 weeks of age [167,168] and, apparently, can contribute to an increase in the level of myoplasmic calcium and muscle cells destruction. It has been established that this may be due to genetically determined rearrangements in MCUC and, in particular, overexpression of MCUb in the inner membrane of organelles, lowering the MCU/MCUb ratio [167]. MCUb overexpression is known to impair the ion-transporting function of the MCUC [169,170], which is apparently also observed in the skeletal muscle mitochondria of *mdx* mice. It should be noted that the expression of other subunits of this complex (MICU1-2 and EMRE) did not change [167]. We also did not reveal any changes in the expression and activity of the Na^+^/Ca^2+^ exchanger, which is responsible for the release of calcium ions from mitochondria and maintaining dynamic transport of the ion in organelles. Interestingly, the use of corticosteroids and, in particular, deflazacort officially approved by the FDA for DMD treatment was accompanied by an improvement in calcium uniport, which may be partly due to the normalization of MCUC composition based on a decrease in the level of the dominant negative MCUb subunit [168].

In the case of heart mitochondria of the same *mdx* mice, we, on the contrary, noted an increase in the efficiency of the Ca^2+^ uniport [171], which, according to the data of Angebault and colleagues, persists up to 3 months of age (adult animals) [172]. This has been shown in other works as well [173,174]. We found that these changes are also accompanied by rearrangements in the MCUC [171]. In cardiomyocytes of *mdx* mice, we noted a significant increase in the content of the channel-forming MCU and the regulatory MICU1 subunits of Ca^2+^ uniporter, while the level of the dominant-negative MCUb subunit is significantly reduced. At the same time, the level of MICU2 and EMRE did not change [171,172]. It is assumed that, in the case of cardiac mitochondria, a significant increase in MCU/MCUb enhances the rate of Ca^2+^ uptake by organelles and the efficiency of ion accumulation in the matrix of *mdx* mice mitochondria [171]. Importantly, the cardiac mitochondria of *mdx* mice also showed an increase in the level of NCLX and the rate of release of calcium ions from the organelles in exchange for Na^+^ (Figure 2) [171]. Activation of calcium transport in the heart mitochondria of young *mdx* mice is also accompanied by an increase in the intensity of oxidative phosphorylation [171,175], which, as we suggest, may determine the adaptation of the heart to an increase in the level of calcium in cardiomyocytes and delay cardiac pathology. It is possible that similar reprogramming is also observed in cardiomyocytes of DMD patients showing signs of metabolic adaptation [176], but this issue needs to be studied. It is also not known what happens to MCUC structure and function in the setting of advanced cardiomyopathy in *mdx* mice or *mdx:utr^−/−^* mice, which exhibit a much earlier onset of heart failure.

It is generally accepted that one of the reasons for the development of mitochondrial dysfunction in DMD, leading to additional calcium overload of muscle cells and induction of muscle cell necrosis, is a decrease in the calcium-buffering capacity of mitochondria and the resistance of organelles to the opening of the calcium-dependent MPT pore, a non-selective protein channel in the inner and outer mitochondrial membranes, which is permeable to molecules less than 1.5 kDa in size (Figure 2) [167,168,177,178,179]. The opening of the MPT pore occurs in the case of excessive accumulation of Ca^2+^ in the mitochondrial matrix, leading to the activation of cyclophilin D (CypD) protein—peptidyl-prolyl cis-trans isomerase, playing an important role in initiating the assembly of the MPT pore channel. Adenine nucleotide translocator (ANT) isoforms and ATP synthase are considered proteins involved in the formation of the pore channel, functioning in different modes [165,166]. Previously, we have suggested that, in the case of DMD, the channel protein may be represented by ANT2 isoform, practically not expressed in healthy skeletal muscles [180] but whose content significantly increased in the skeletal muscles of dystrophin-deficient *mdx* mice [167]. It should be noted that the level of ATP synthase did not change or even decreased [167]. It is also important to note a decrease in mRNA expression and the level of CypD protein in the skeletal muscles of *mdx* mice, which may be an attempt to adapt to organelle calcium overload and early opening of the MPT pore, but this does not have a preventive effect [167,181]. However, genetic inactivation or pharmacological inhibition of the MPTp regulatory protein CypD by specific suppressor cyclosporin A (CsA), and its non-immunosuppressive analog alisporivir (or Debio025) or isoxazoles (as TR001), resulted in improved mitochondrial function including the ability of mitochondria to accumulate calcium ions [181,182,183,184,185,186]. In the case of alisporivir we also noted an improvement in calcium uniport in the skeletal muscle mitochondria of *mdx* mice, which may be due to the effect of this agent on the ratio of MCUC channel subunits [187]. The positive effect of these agents was also noted on biopsy specimens of patients suffering from DMD [183].

On the contrary, in the case of heart mitochondria of *mdx* mice, we found an increase in calcium capacity, which is observed in young animals and indicates an increase in resistance to MPT pore induction [171,175] and can also be considered as an adaptive mechanism that compensates for SR dysfunction. A high level of mitochondrial calcium persists in more mature animals, despite a decrease in membrane potential and the development of oxidative stress [179]. However, we did not note changes in the level of putative MPT pore proteins (CypD, ANT1 and ANT2, ATP synthase, and VDAC1) in *mdx* hearts and, apparently, this pattern is associated with other factors, in particular, with an increase in the microviscosity of mitochondrial membranes in the hearts of *mdx* mice [171], which has been shown to increase the resistance of organelles to ANT-mediated MPT pore opening [188]. However, in this case, the use of alisporivir also led to the restoration of some parameters of the functioning of the heart mitochondria [175]. It should also be noted that, similarly to MCUC and NCLX, there are currently no data on the activity and structure of the MPT pore in advanced cardiomyopathy (both in model animals and biopsy specimens).

Concluding the topic of MPT pore, we would like to note that we have not found data on the role of the lipid pore induced by long-chain fatty acids [165] in the recycling of Ca^2+^ ions through the mitochondrial membrane. However, one could assume that DMD may be accompanied by changes in the mechanisms and activity of the induction of this phenomenon. Indeed, DMD is well known to be accompanied by an increase in the activity of phospholipase A_2_ [110], which is capable of hydrolyzing phospholipids and thereby enriching membranes, including mitochondrial ones, with free fatty acids and, as a result, inducing their fatty acid/calcium-dependent permeabilization.

Our recent works highlight the possible role of mitochondrial potassium channels in the development of DMD [189,190]. The dysregulation of potassium channels is known to play an important role in the development of myopathies and, in particular, the dilated cardiomyopathy of various etiologies [95,96], which is also characteristic of Duchenne muscular dystrophy. We have recently shown a reduction in mitochondrial potassium transport in the skeletal muscles of *mdx* mice, accompanied by a decrease in the expression of the VEDEC splice variant from *Kcnma1* gene encoding the calcium-activated potassium channel (BK_Ca_) in the inner mitochondrial membrane (Figure 2) [189]. In this case, the activator of BK_Ca_ channel NS1619 normalized the transport of K^+^ and the level of the ion in the skeletal muscle mitochondria of *mdx* mice, as well as the expression of BK_Ca_, which was accompanied by an improvement in the morphology of mitochondria, an increase in resistance to MPT pore opening, and also generally had a positive effect on the state of the skeletal muscles of animals [189]. The normalization should also be noted of potassium transport in the skeletal muscle mitochondria of *mdx* mice treated with uridine [190], a precursor of uridine 5’-diphosphate (UDP), which is an activator of the mitochondrial ATP-dependent potassium channel (mitoK_ATP_) [191] suggesting an important role of the latter in the disruption of mitochondrial potassium transport in DMD. However, we do not exclude that the effect of uridine in this case may be due to its modulation of cellular function and energy metabolism [192]. Nevertheless, recent data also point to the importance of potassium channels in the development of mitochondrial dysfunction and dysregulation of muscle cell ion homeostasis in DMD, and this requires further study.

### 4.3. SR–Mitochondria Axis and MAM Contacts in DMD

The SR membrane and the outer membrane of adjacent mitochondria are known to form common areas, the so-called MAM (mitochondria-associated membranes) contacts that are necessary for correct communication between these organelles. Currently, MAM have been linked to metabolic regulation, autophagy, aging, senescence, and ROS production [193]. One of the main functions of MAM is the transport of calcium from SR to mitochondria, which makes it possible to finely regulate the activity of mitochondria and their role in the implementation of physiological and pathological signals in the cell [165]. The protein composition of MAM contacts is dynamic and depends on the conditions and metabolic activity of the cell and, according to proteomic data, includes more than 1000 proteins [194]. IP_3_R is responsible for the release of calcium from the SR in this region. In turn, calcium released from the SR freely penetrates into mitochondria through the VDAC of the outer membrane of these organelles. The interaction between IP_3_R and VDAC is provided by the GRP75 (glucose-regulated protein 75) chaperone protein, which both physically binds IP_3_R to VDAC into a complex and ensures their functional coupling facilitating the entry of Ca^2+^ into mitochondria [195]. IP_3_R expression is shown to be altered in both dystrophic human and dystrophic mouse muscle cells (Figure 2) [196,197,198]. In particular, IP_3_R1-GRP75 and IP_3_R1-VDAC1 interactions were significantly decreased in the diaphragm of *mdx* mice compared to wild-type animals [199]. In this case, tauroursodeoxycholic acid suppressing reticulum stress was found to increase SR/ER–mitochondria physical contact and improved *mdx* muscle contractile function [199]. Pharmacological blockade by xestospongin or genetic inactivation of IP_3_R has also been shown to decrease basal Ca^2+^ levels, correct Ca^2+^ release upon stimulation, reduce mitochondrial Ca^2+^, and restore muscle function in *mdx* mice [200,201,202]. All this confirms the important role of SR/mitochondria communications and MAM contacts in the development of calcium dysregulation in DMD. However, it is important to note that *mdx* mouse cardiomyocytes, along with the above-described increase in MCU activity, demonstrate an increase in the number of IP_3_R1–VDAC1 contacts points [172] confirming our information about the adaptive capabilities of this organ in terms of the regulation of calcium homeostasis.

## 5. Contribution of SR and Mitochondrial Channels to the Development of DMD

The existing data allow us to conclude that the SR–mitochondria axis is unable to perform its function as an intracellular calcium depot leading to further calcium overload of the myoplasm and causing the development of the pathological processes indicated in Section 3. Firstly, this is due to a decrease in the efficiency of calcium uptake in the SR through SERCA and, conversely, to excess leakage through RyR. Secondly, it is due to a decrease in the efficiency of calcium transfer through MAM contacts, which, along with the suppression of ion uniport through the MCU, does not allow pumping excess calcium into mitochondria. Thirdly, it is due to a decrease in the ability of mitochondria to retain calcium ions in the matrix due to the active induction of the MPT pore. Pharmacological or genetic modulation of at least one of these pathways or sarcolemmal channels contributes to the alleviation of pathology (Table 1).

Along with this, it can be seen that calcium overload is accompanied by a violation of other functions of the SR and mitochondria, which are multi-functional organelles. In particular, SR controls secretory protein folding and modification, sterol and fatty acid biosynthesis, and stress signaling [154]. These processes appear to be also impaired in DMD (Figure 2); in particular, the skeletal muscles of *mdx* mice show activation of the unfolded protein response (UPR) limiting accumulation of unfolded and misfolded proteins [199,204]. The muscles of DMD patients also show an increase in the level of the UPR marker (GRP78 chaperone). De-stressing the SR with tauroursodeoxycholic acid has been shown to decrease the UPR response in the muscles of *mdx* mice [199].

Currently many reviews have been published on mitochondrial dysfunction in DMD, demonstrating a significant contribution to the dramatic progression of the pathology [12,14,26,114,115,153]. Along with the regulation of calcium homeostasis (although the role of these organelles is not so significant compared to SR), mitochondria produce the majority of ATP required for muscle contraction and other processes, as well as generate ROS, and they are involved in the regulation of cell death pathways. Calcium overload and accumulation of intracellular ROS and RNS have a direct impact on the function of these organelles. Indeed, skeletal muscle mitochondria of *mdx* mice show a decrease in the efficiency of oxidative phosphorylation and ATP synthesis [167,168,177,178,181,205], which is accompanied by a change in the level of electron transport chain (ETC) complexes and the efficiency of their functioning, as well as a decrease in the membrane potential of organelles (Figure 2) [168,177,178,181,205]. A decrease in the efficiency of ATP synthesis is also shown on biopsy specimens of DMD patients [176,183]. Dystrophin-deficient animals also show a reduction in some important mitochondrial inner membrane exchangers and, in particular, ANT1, which is responsible for the release of ATP from mitochondria in exchange for ADP [159,167]. Interestingly, according to some hypotheses, these rearrangements may be due to a decrease in ATP demand by the dysfunctional contractile apparatus of skeletal muscles [115]. Mitochondrial dysfunction is also accompanied by a reduction in organelle biogenesis, mitophagy, and removing damaged mitochondria, as well as changes in the dynamics (fusion and fission) of organelles [181,206,207]. An important contribution of dysfunctional mitochondria to ROS overproduction is noted [178], which, along with a decrease in resistance to MPT pore opening, promotes the release of protein factors from organelles that initiate muscle fiber necrosis, and this is also prevented by pharmacological or genetic blockade of the MPT pore [181,182,183,184,185,186]. Our latest data show that a decrease in the efficiency of potassium ion transport and the activity of potassium transporters may also cause ROS overproduction (Figure 2) and active opening of the MPT pore in the mitochondria of *mdx* mice, and potassium channel activators improve mitochondrial function and muscle tissue condition [189,190].

Surprisingly, the heart of young *mdx* mice shows an activation of calcium transport and ion buffering, which manifests itself at least as an increase in SR and mitochondrial contact interactions (MAM contacts), an increase in calcium uniport and resistance to the MPT pore opening, and a more efficient operation of NCLX [171,172,175]. This seems to temporarily compensate for the dysfunction of the cardiomyocyte SR and contributes to the later development of arrhythmia and other electrophysiological abnormalities reflecting the function of the cardiac contractile apparatus. This pattern is also characteristic of the human variant of DMD demonstrating a late development of cardiomyopathy compared to skeletal muscle dysfunction. This is also accompanied by an increase in the respiratory activity of organelles, which is noted in *mdx* mice [171,175]. In the case of young DMD patients, a healthy-like metabolic status and mitochondrial respiratory activity was also found [176]. However, in later stages, model animals and DMD patients show a significant decrease in mitochondrial function, which is also accompanied by abnormal organelle morphology [179].

## 6. The Summary

Dysregulation of muscle cell ion channels is one of the most important consequences of the loss of the dystrophin protein and the functional integrity of DAPC. This is manifested both in the disruption of the functioning and regulation of the channels of the sarcolemma and the channels of intracellular organelles responsible for maintaining ion homeostasis. Nevertheless, given that DMD does not demonstrate embryonic lethality, one could assume that in some cases there is an adaptive reprogramming of many systems of the organism, including the expression and regulation of ion channels. This is especially actual in the case of cardiac tissue and corresponds to the delayed development of cardiomyopathy, which is typical both for humans and some model organisms. Currently known DMD-induced cardiospecific changes in ion channels are highlighted in Figure 2 (by the heart symbol). Unfortunately, these rearrangements often look insufficient and do not lead to an improvement in the functional characteristics of ion transport.

## 7. Clinical Implications

Pharmacological or genetic modulation of the ion channels of the sarcolemma and organelles in some cases leads to an improvement in the state and function of muscle tissue and the quality of life of the experimental object. However, it should be noted that many of these results are obtained in model animals and are not confirmed in clinical trials. This requires both the creation of more adequate pathology models and careful interpretation of the data. Despite this, the body of evidence suggests that ion channel targeting may be a promising approach for the secondary therapy of DMD and other muscular dystrophies.

## Figures and Tables

**Table 1:** Ion channels and membrane defects in DMD and their pharmacological modulators.

Target	Drug	Mechanism of Action	Models Used	References
Membrane microtears	Poloxamer 188	Seals microtears in the sarcolemma	Canine model, *mdx,* and *mdx/utrn^–/–^* mice	Townsend et al. [34] Houang et al. [35] Markham et al. [36]
TRPC3	Pyr10	Prevents calcium overload	DMD rats	Creisméas et al. [51]
TRPC6	BI 749327	Prevents calcium overload	*mdx/utrn^–/–^* mice	Lin et al. [50]
L-type Ca_V_	Diltiazem, verapamil, nifedipine	Prevent calcium overload	*mdx* mice	Matsumura et al. [59] Altamirano et al. [60]
P2X7	Coomassie Brilliant Blue G 250, oxidized ATP, suramin	Prevent calcium overload	*mdx* mice, BIO14.6 hamsters	Sinadinos et al. [63] Gazzerro et al. [64] Taniguti et al. [65] Iwata et al. [85]
STIM1-Orai1	AnCoA4, CM4620, and GSK7975A	Reduce Orai1-induced Ca^2+^ influx into the myoplasm	DMD-patient-derived myotubes	Uchimura et al. [75]
Na_V_1.4	Tetrodotoxin	Prevents sodium overload	*mdx* mice	Hirn et al. [83]
NHE	Cariporide, 5-(N-ethyl-N-isopropyl)-amiloride, rimeporide	Prevent sodium overload	*mdx* mice, BIO14.6 hamsters, DMD patients	Iwata et al. [85] Previtali et al. [87]
SERCA1 or SERCA2a	AAV.SERCA	Overexpresses SERKA and improves calcium uptake by SR	*mdx* and *mdx/utrn^–/–^* mice	Goonasekera et al. [123] Mazala et al. [124] Wasala et al. [126]
SLN	AAV.SLN	Downregulates SLN and activates SERKA	*mdx/utrn^–/–^* mice	Viner et al. [109]
RyR	Rycals	Improve binding of calstabin to RyR and prevent RyR Ca^2+^ leak	*mdx* mice, DMD-patient-derived myotubes and cardiomyocytes	Capogrosso et al. [151] Barthelemy et al. [152] Meyer et al. [203]
VDAC	VDAC peptide	Prevents the opening of VDAC and rescues mitochondrial membrane potential	*mdx* mice	Viola et al. [160]
MPT pore	CsA, alisporivir, isoxazoles	Desensitize MPT pore to activation by calcium	*mdx* mice, Zebrafish model, DMD-patient-derived myotubes	Dubinin et al. [181] Millay et al. [182] Schiavone et al. [183] Reutenauer et al. [184] Wissing et al. [185] Stocco et al. [186]
mitoBK_Ca_	NS1619	Activates potassium transport into mitochondria	*mdx* mice	Dubinin et al. [189]
mitoK_ATP_	Uridine (precursor of uridine 5’-diphosphate (UDP)	Activates potassium transport into mitochondria	*mdx* mice	Dubinin et al. [190]
IP_3_R	Xestospongin	Inhibits IP_3_R and prevents calcium overload	*mdx* mice	Altamirano et al. [200] Mijares et al. [201]

## Data Availability

Not applicable.

## Abbreviations

ANT	Adenine nucleotide translocator
BK_Ca_	High conductance Ca^2+^-activated K^+^ channel
Ca_V_	Voltage-gated calcium channel
CypD	Cyclophilin D
DAPC	Dystrophin-associated protein complex
DMD	Duchenne muscular dystrophy
DWORF	Dwarf open reading frame
GRP75	Glucose-regulated protein 75
IP_3_R	Inositol 1,4,5-trisphosphate receptor
K_ATP_	ATP-sensitive potassium channel
Kir	Inward-rectifier potassium channel
MAM	Mitochondria-associated membranes
MCUC	Mitochondrial calcium uniporter complex
MLN	Myoregulin
MPT	Mitochondrial permeability transition
Na_V_	Voltage-gated sodium channel
NCLX	Na^+^-Ca^2+^-Li^+^ exchanger
NCX	Na^+^/Ca^2+^ exchanger
NHE	Na^+^–H^+^ exchanger
NOS	Nitric oxide synthase
PLN	Phospholamban
RNS	Reactive nitrogen species
ROS	Reactive oxygen species
RyR	Ryanodine receptor
SERCA	Sarco/endoplasmic reticulum Ca^2+^-ATPase
SLN	Sarcolipin
SOCE	Store-operated Ca^2+^ entry
SR	Sarcoplasmic reticulum
STIM1	Stromal interaction molecule
TRIC	Trimeric intracellular cation channel
TRPC	Transient receptor potential channel
TRPV2	Transient receptor potential vanilloid type 2
UPR	Unfolded protein response
VDAC	Voltage-dependent anion channel
